# Effects of *Solanum tuberosum* L. ointment on second-degree burns in mice

**DOI:** 10.14202/vetworld.2023.2440-2445

**Published:** 2023-12-16

**Authors:** Carmen R. Silva-Correa, Galy P. Rosas-Cruz, Abhel A. Calderón-Peña, Víctor E. Villarreal-La Torre, Cinthya L. Aspajo-Villalaz, Julio A. Castañeda-Carranza, Deivy Y. Dionicio-Rosado, Ricardo M. Gómez-Arce, Cristhian N. Rodríguez-Silva, Jorge Del Rosario-Chávarri, José L. Cruzado-Razco

**Affiliations:** 1 Department of Pharmacology, Faculty of Pharmacy and Biochemistry, National University of Trujillo, Peru; 2 Department of Biological Chemistry and Animal Physiology, Faculty of Biological Sciences, National University of Trujillo, Peru; 3 Professional Department of Statistics, Faculty of Physical Sciences and Mathematics, National University of Trujillo, Peru.

**Keywords:** burn healing, herbal medicine, histopathology, ointment, potato, skin, *Solanum tuberosum* L

## Abstract

**Background and Aim:**

Potato (*Solanum tuberosum* L.) is mainly characterized by its antioxidant and healing properties. Therefore, this study aimed to evaluate the effects of an ointment based on *S. tuberosum* L. “papa tumbay” on burns induced in Balb/c mice (*Mus musculus*).

**Materials and Methods:**

The experimental animals were divided into four groups (n = 5/group) 48 h before second-degree burns were inducted. After epilating the loin areas of the mice and anesthetizing them with ketamine/xylazine (80 mg/kg/10 mg/kg) through intraperitoneal (i.p.) route, a round metal rod (0.7 cm in diameter) was placed on the depilated skin at a temperature of 100°C for 5 s. Group I was not given any treatment, Group II was treated with silver sulfadiazine (1%), and the other two groups (III and IV) were treated with the ointment formulated based on *S. tuberosum* L. “papa tumbay” at 1% and 2%, respectively. After performing the treatment for 21 days, the mice were euthanized using i.p. sodium pentobarbital (185 mg/kg) to obtain skin samples. The samples were preserved in 10% neutral-buffered formalin and subjected to histopathological analysis.

**Results:**

We found statistically significant differences in the histopathological sections between the groups (p < 0.05). The abundant collagen and fibroblasts observed in the direction of the dermis in Groups III and IV indicate that the phytoconstituents present in the potato might promote the healing of the second-degree burns until day 21 of treatment.

**Conclusion:**

Our findings showed that the ointments based on the ethanolic extracts of *S. tuberosum* L. “papa tumbay,” especially the 2% ointment, might accelerate the healing of second-degree burns induced in Balb/c mice.

## Introduction

Burn injuries, a global public health problem [1], commonly result from exposure to heat and chemicals [2]. These cause several alterations ranging from local erythema to total tissue destruction. The degree of burn depends on the temperature, contact time, and the affected anatomical region of the skin. It is classified by severity level into Grades I, II, and III [3]. The pathophysiological process of burn healing is complex and dynamic and includes three interrelated phases of hemostasis inflammation, proliferation, and remodeling [4]. Extensive tissue damage during burns affects angiogenesis, collagen reorganization, and granulation tissue formation, and induces free radical-mediated damage, delaying tissue repair [5].

After a burn, the skin becomes vulnerable to infections by Gram-negative bacteria (*Escherichia coli*, *Pseudomonas aeruginosa*, and *Proteus* species) and Gram-positive bacteria (*Staphylococcus aureus*). These burn wound infections cause the formation of bedsores and blisters on the skin, which delay the wound-healing process [6]. Therefore, recovery from a burn wound requires prolonged hospitalization, expensive medication, and extensive rehabilitation [5]. Conventional treatment for burns includes the application of a topical silver sulfadiazine drug, which has broad-spectrum antimicrobial activity and inhibits the growth of bacteria and fungi, mainly *Candida albicans* [7].

The main challenges in treating burns are achieving rapid wound healing and preventing infections [8]. This has led researchers to seek therapeutic alternatives from natural sources, such as plants, that have been described in traditional Chinese, Indian, and Iranian medicine and have been scientifically evaluated [3]. Potato (*Solanum tuberosum* L.) is a food crop native to South America and cultivated worldwide due to its appreciable nutritional benefits in humans [9]. Potato is an excellent source of Vitamin C and other biologically active substances, such as polyphenols and flavonoids, commonly described as antioxidants [10] and located mainly in the potato skin. A previous study by Rosas-Cruz *et al*. [11] showed the healing effects of an ointment based on the ethanolic extract from *S. tuberosum* L. “papa tumbay” peels on a mouse model for wounds.

Therefore, this study aimed to evaluate the effects of an ointment based on *S. tuberosum* L. on second-degree burns induced in mice.

## Materials and Methods

### Ethical approval

The study was approved by the Ethics Committee of the School of Pharmacy and Biochemistry of the Universidad Nacional de Trujillo with the document COD. N°: P-005-2020/CEIFFYB.

### Study period and location

The study was conducted from December 2020 to March 2021. All processes were performed in the Toxicology Laboratory, School of Pharmacy and Biochemistry, Universidad Nacional de Trujillo.

### Botanical materials

We collected 5 kg of *S. tuberosum* L. “papa tumbay” tubers from a village in Villamaria, Carabamba district, Julcán province, La Libertad, Peru. A single *S. tuberosum* L. specimen was taken to the Herbarium Truxillense of the Universidad Nacional de Trujillo for identification and taxonomic verification (code N°59729).

### Experimental animals

We randomly selected 20 healthy adult Balb/c mice, including both males and females, with average body weights of 30–35 g and 3–4 months of age for the experiments. The animals were acclimatized in the bioterium of the faculty of pharmacy and biochemistry of the Universidad Nacional de Trujillo at temperatures between 20°C and 22°C with a light and dark cycle of 12/12 h. They received standard feed and water *ad libitum*.

### Preparation of ethanolic extract

Ethanolic extract was prepared as per the method described by Rosas-Cruz *et al*. [11]. After washing the tubers and removing the skin, the pulp was cut into small pieces, placed in an amber jar with 96% alcohol, and macerated for 72 h with daily manual stirring. Then, it was filtered with cotton cloth and kept in the oven at 40°C for 48 h to obtain the dry extract, which was preserved in an amber jar and refrigerated until use.

### Preparation of the ointment based on *S. tuberosum* L.

Ointment based on *S. tuberosum* L. was prepared as per the method described by Rosas-Cruz *et al*. [11]. The ointments were prepared by mixing the 1% and 2% ethanolic extracts obtained from *S. tuberosum* L. “papa tumbay” peels with components, including lanolin and petroleum jelly. Finally, after packaging and labeling, the ointments were stored at 20ºC until use.

### Experimental groups

The Balb/c mice were randomly distributed (simple randomization) into four groups (n = 5 mice/group): Group I (negative control): Mice with second-degree burns left untreated; Group II (positive control): Mice with second-degree burns treated topically once a day with 1% silver sulfadiazine for 21 days; and Group III and IV: mice with second-degree burns treated topically with 1 and 2% ointment, respectively, of *S. tuberosum* L. once a day for 21 days.

### Induction of second-degree burns

Second-degree burn was induced as per the method described by Kaboutari *et al*. [12]. The loin areas of the mice were epilated 48 h before the second-degree burns were induced. After anesthetizing with ketamine (80 mg/kg) and xylazine (10 mg/kg) intraperitoneally (i.p.), the second-degree burns were induced using a hot metal rod (0.7 cm in diameter) placed directly on the depilated skin for 5 s at 100°C. After inducing the burns, each animal was placed in a separate cage.

### Evaluation of the healing process

We evaluated the healing time of the second-degree burns as described by Javanmardi *et al*. [3] and obtained the respective photographic controls throughout the experimental period. The measured area of the second-degree burn using a vernier and the percentage of healing was calculated with the formula:


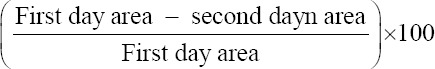


### Histopathological analysis

Histopathological analysis was performed as per the method described by Silva-Correa *et al*. [13]. The animals were euthanized using i.p. sodium pentobarbital (185 mg/kg). Skin samples were obtained by making a 1.5 cm × 1.5 cm incision around the scar. Samples were preserved in sterile flasks with 10% formaldehyde solution for 8 days, and then, 3-5 μm parts were selected and fixed in paraffin. Samples were stained with hematoxylin-eosin and observed under a light microscope (Motic, España) at ×400.

### Statistical analysis

The results were analyzed with the R-4.1.1 program for Windows® (https://cran.r-project.org) using box and whisker plots, analysis of variance in a completely random design, and Tukey’s *post hoc* test. Statistical significance was considered at p < 0.05.

## Results

### Evaluation of the healing effect

**Figure-1:**
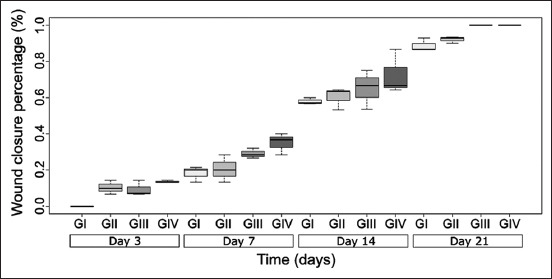
Percentage of healing of second-degree burns induced in *Mus musculus* Balb/c. in the experimental groups. p < 0.05, n = 5.

The percentage of burn healing was evaluated using the wound closure parameter to determine the progress in healing. The percentages of wound closure on day 21 for groups III and IV (treated with 1% and 2% *S. tuberosum* ointment, respectively) were significantly different than that in Groups I (control) and II (treated with silver sulfadiazine) (p < 0.05). Of all groups, the greatest effect was observed in the group that was treated with the 2% ointment (Figure-1).

### Histopathological analysis

**Figure-2:**
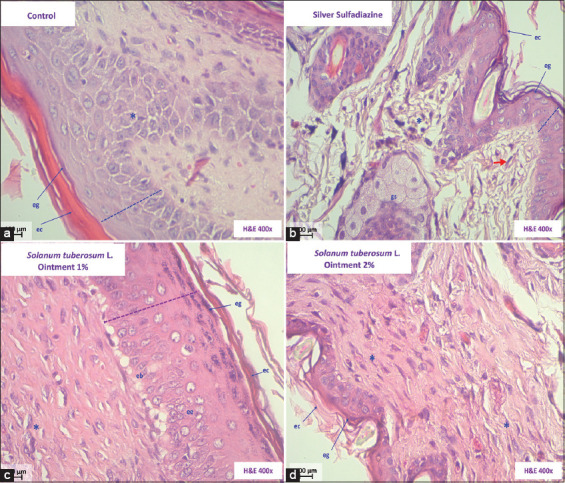
Histopathological skin sections of *Mus musculus* Balb/c. (a) Group I (Control). (b) Group II (silver sulfadiazine). (c) Group III (Ointment *Solanumtuberosum* 1%). (d) Group IV (Ointment *S. tuberosum* 2%). Keratinous stratum corneum (CE), Stratum granularis (EG), Sweat glands (GS), Stratum spinosum (EE), Basal cells (EB), and fibroblasts (*) (Hematoxylin and Eosin, 400×).

Several histopathological changes were observed in the skin of mice in Group I (control), including the presence of a crust or eschar with connective tissue containing fibroblasts, low activity of the stratum spinosum and basale cells to restore the dermal papillae, and dense collagen-rich areas in the lower right area. These findings correspond to the physiological progressive healing process (Figure-2a). In Group II (treated with silver sulfadiazine), we observed a slight arrangement of the epidermis to restore intradermal papillae, suggesting the onset of re-epithelialization and sweat gland activity (Figure-2b). The healing activity observed in Groups III and IV that received the ointments of *S. tuberosum* L. “papa tumbay” on day 21 after treatment indicated recovery of the epidermal stratum, granular, spinous, and basale cells. In the dermis, abundant collagen and fibroblast activity arranged in a horizontal position were observed. Moreover, this effect was greater in Group IV (Figures-2c and d).

## Discussion

In this study, we evaluated the effects of ointments based on the ethanolic extracts of *S. tuberosum* L. “papa tumbay” peels on the healing of second-degree burns induced in mice.

Burns are common traumatic injuries that cause local tissue damage and systemic mediator-induced response. Evidence of both local and systemic oxidative changes manifested by increased free radical activity and lipid peroxidation exists. At the same time, burns cause a notable decrease in total antioxidant status and a reduction in antioxidant scavenging capacity compared to normal tissues. Excess free radicals overwhelm the natural radical blocking or scavenging mechanisms, producing oxidative stress. Therefore, addressing this condition with antioxidants might be a promising approach to improve the outcomes of burns [14].

Our results showed that treatments with *S. tuberosum* L. can significantly influence the healing process of burns. The area of the second-degree burn decreased in a statistically significant manner in the groups that received *S. tuberosum* L. ointments compared to the negative and positive control groups (p < 0.05) (Figure-1). Moreover, an ointment containing 2% *S. tuberosum* L. extract had a remarkable healing effect on second-degree burns with decreased recovery time. This effect can be attributed to the phenolic components and antioxidant properties of the potato peel ethanolic extracts [11]. Potato periderm (skin) protects the potato tuber against phytopathogens and contains numerous secondary metabolites, including phenolic compounds [15]. These phenolic compounds, including caffeic acid, vanillic acid [16], hydroxycinnamic acid, and flavonoids, contribute to the antioxidant activity in potatoes [15].

Figure-2 shows the histopathological characteristics of the skin samples obtained from the experimental groups after 21 days of treatment. In the negative control group (Figure-2a), we observed that in the epidermis had a crust or eschar with connective tissue containing fibroblasts. The dotted line denotes the slight activity of the stratum spinosum and basale cells to restore the dermal papillae. Further, collagen-rich areas can be seen in the lower right area. These findings correspond to the physiological healing process, which begins with trauma and usually ends with the formation of scars [17, 18].

Wound healing is a semi-reversible process involving processes, such as inflammation, proliferation, and remodeling, and mediators, including epidermal growth factor and fibroblast growth factor 2 [19]. The fibroblasts actively move into the wound area to support the proliferating tissue. They also produce collagen, enabling the formation of granulation tissue that covers the wound [20].

In the positive control group that received silver sulfadiazine (Figure-2b) for 21 days, we observed a slight arrangement of the epidermis to restore intradermal papillae, indicating the onset of re-epithelialization and sweat gland activity [21–23]. Silver sulfadiazine ointment is commonly used to treat burn wounds as it prevents wound infection by inhibiting microbial growth. However, this treatment is time-consuming and associated with issues, such as pain and discomfort for patients [24]. Moreover, drugs with a high content of silver ions can bind to the RNA in epithelial cells, hindering the regeneration of epithelial cells. Therefore, this drug can potentially irritate wounds and is unfavorable for wound repair [25].

The groups that were treated with the ointments containing 1% and 2% extracts of *S. tuberosum* L., “papa tumbay” (Figures-2c and 2d) exhibited improved active recovery of the epidermal strata, including corneal, granular, spinous, and basale cells, compared to the negative and positive control groups. The cellular elements were restored in the epidermis, while abundant collagen and fibroblast activity arranged horizontally to the epidermis was observed in the dermis. The best outcome was observed for re-epithelialization and granulation tissue formation with well-formed and horizontally oriented collagen fibers. Tissue remodeling and repair requires collagen, matrix proteins, and fibroblasts during the proliferative phase. The proliferating fibroblasts in the wound area produce various components necessary for wound repair, such as collagen, proteoglycan, fibronectin, hyaluronan, and matrix proteins [26]. Our findings suggested that treatment with *S. tuberosum* L. “papa tumbay” ointment supported fibroblast proliferation and collagen formation in the wound tissue, accelerating epithelialization. This effect was more pronounced in the group treated with the 2% ointment.

Reactive oxygen species are produced during wound healing and have a dual role. In an acute manner, they can stimulate the division and migration of endothelial cells and fibroblasts, which promotes the reformation of blood vessels and the formation of new extracellular matrix and collagen (ECM), respectively. This further promotes the proliferation and migration of keratinocytes. However, excessive ROS can contribute to the formation of hypertrophic scars [27, 28].

Burns are associated with increased free radical activity and lipid peroxidation. The enzyme xanthine oxidase is a source of oxidative stress during burn trauma, producing superoxide anions and hydrogen peroxide. Contrastingly, activated neutrophils can also produce superoxide anions [29]. Since phenolic compounds are powerful antioxidants, *S. tuberosum* can be used in the treatment of burns.

Although potato peels are often discarded, reports have shown the presence of phenolic compounds and flavonoids in these parts, indicative of their antioxidant, anti-inflammatory, and antibacterial activities [11]. These properties can act synergistically to enable wound healing. Sarandy *et al*. [30] showed the healing effects of topical administration of various compounds isolated from plant extracts, including polysaccharides from *S. tuberosum*, on skin wounds in murine experimental models. Rosas-Cruz *et al*. [11] showed that an ointment based on *S. tuberosum* L. “papa tumbay” had a healing effect on wounds induced in mice, which was more pronounced in ointments containing 2% extract. They also reported the antioxidant activity of the ethanolic extracts from *S. tuberosum* L. “papa tumbay” peels. Considering that high ROS levels complicate wound healing, the healing activity of *S. tuberosum* L. might be attributed to its antioxidant activity [31].

*Solanum nigrum* is another potato species that has been shown to have a healing effect on burns. A 20% hydroalcoholic extract of *S. nigrum* improved a few wound healing parameters, such as collagen deposition and inflammation. It also shows an inhibitory effect on *S. aureus*. Therefore, this extract can also treat burns [24].

The phytochemical constituents in the ethanolic extracts of *Solanum xanthocarpum* leaves were qualitatively identified and their wound healing activity was evaluated. Topical application of these extracts showed significant wound healing activity in rat excision and incision wound models. In this study, five groups of animals (n = six rats/group) were treated with topical application of 10% *S. xanthocarpum* leaf extracts (w/v) in saline solution using silver sulfadiazine ointment as a standard. The results showed that when applied topically, this extract reduced the epithelialization period from 25.3 ± 0.23 to 19.75 ± 0.28 days (as seen in the control and ethanol extract-treated groups, respectively). Moreover, the scar area markedly decreased from 53.88 ± 0.42 to 37.76 ± 0.17 mm^2^ in the control and ethanol extract-treated group, respectively. A significant increase in tensile strength and hydroxyproline content of the plant extract was also observed and compared with control and silver sulfadiazine [32].

The phytoconstituents in potatoes might promote and accelerate the healing process of second-degree burns until day 21 of treatment. The main metabolites found in potatoes are polyphenols, flavonoids (including rutin), and alkaloids (including solanine) [33, 34]. Potato peels also contain low amounts of phenolic acids, mostly composed of chlorogenic acids and other phenols, such as caffeic acid, gallic acid, and protocatechuic acid [9]. Chlorogenic acid has been shown to enhance capillary density and promote collagen production. Along with its free radical scavenging properties, it exerts anti-oxidant and anti-inflammatory on the metalloproteinases in the ECM of the injured tissues during second-degree burns [35, 36].

## Conclusion

The phytoconstituents present in the potato might accelerate the healing process of second-degree burns until day 21 of treatment. The ointments based on the ethanolic extract of *S. tuberosum* L. “papa tumbay” can be used to treat second-degree burns induced in mice, with the 2% ointment being more effective.

## Authors’ Contributions

CRS and JLC: Conceptualization, drafted the manuscript, and preparation of extract. GPR and JDR: Collected the plant species and entered them in the herbarium. VEV: Validation, supervision, and formal analysis. JAC, DYD, and RMG: Performed the statistical analysis and the prepa­ration of images. AAC, CNR, and CLA: Performed organ harvesting for histopathological analysis, kept the animals during the investigation, and administered treatments. All authors have read, reviewed, and approved the final manuscript.
